# Short-term corticosteroid therapy in aspiration pneumonitis complicated by acute respiratory distress syndrome: A case report

**DOI:** 10.1097/MD.0000000000047816

**Published:** 2026-02-20

**Authors:** An Le-Hoang, Khoa Nguyen-Dang, Huy Tran-Dinh, Quoc-Khanh Tran-Le, Ngoc Duong-Minh, Hanh-Duyen Bui-Thi

**Affiliations:** a Department of Internal Medicine, School of Medicine, University of Medicine and Pharmacy at Ho Chi Minh City, Ho Chi Minh City, Vietnam; b Department of Pulmonary Medicine, Cho Ray Hospital, Ho Chi Minh City, Vietnam; c Department of Intensive Care, University Medical Center Ho Chi Minh City, University of Medicine and Pharmacy at Ho Chi Minh City, Ho Chi Minh City, Vietnam.

**Keywords:** acute respiratory distress syndrome, anesthesia, aspiration pneumonitis, corticosteroid, Mendelson syndrome

## Abstract

**Rationale:**

Aspiration of gastric contents (Mendelson syndrome) can cause chemical pneumonitis and quickly progress to acute respiratory distress syndrome (ARDS). Evidence for corticosteroids in this setting is limited, and guidelines are unclear. We describe a postpartum case of aspiration pneumonitis evolving to ARDS that improved after a short course of methylprednisolone.

**Patient concerns:**

A 27-year-old woman developed severe dyspnea, chest tightness, and hypoxemia after a cesarean section performed under general anesthesia.

**Diagnoses:**

On arrival at Cho Ray Hospital, she was tachypneic with bilateral crackles and a PaO_2_/FiO_2_ ratio of 78.4. Chest radiography showed diffuse bilateral infiltrates, and bronchoscopy revealed edematous, secretion-free airways, findings consistent with aspiration pneumonitis progressing to early ARDS.

**Interventions:**

The patient was intubated, treated with lung-protective mechanical ventilation, broad-spectrum antibiotics, supportive care, and intravenous methylprednisolone at 1 mg/kg/day for 2 days.

**Outcomes:**

Oxygenation indices rose rapidly after steroid initiation, allowing stepwise ventilator weaning. She was extubated on day 5 and discharged on day 10 without respiratory symptoms, and postpartum recovery was uneventful.

**Lessons:**

Mendelson syndrome can progress within hours to severe ARDS even in otherwise healthy postpartum patients. Early airway protection with lung-protective ventilation, consideration of early bronchoscopy when feasible, and stewardship-based empiric antibiotics with de-escalation are important. A carefully monitored, time-limited corticosteroid trial may be considered in selected cases of chemical aspiration-related moderate to severe ARDS, but treatment should be individualized with close reassessment.

## 1. Introduction

Aspiration in respiratory diseases refers to the entry of substances from the oropharynx or stomach into the larynx and lower airways.^[1]^ Depending on the chemical nature, volume aspirated, and host response, this can lead to various syndromes such as chemical pneumonitis, bacterial aspiration pneumonia, post-obstructive pneumonia, exogenous lipoid pneumonitis, or chronic interstitial fibrosis.^[1,2]^ Sterile gastric contents cause chemical pneumonitis and are distinct from aspiration pneumonia, which involves inhalation of bacteria from oropharyngeal secretions.^[3]^ In addition to standard treatments such as oxygen therapy, fluid replacement, prophylactic anticoagulation, airway protection, and respiratory physiotherapy and rehabilitation, the use of other interventions like prophylactic antibiotics and corticosteroids in aspiration pneumonitis remains controversial and lacks consensus regarding their efficacy.^[1,4–7]^ Although several pathophysiological mechanisms suggest a potential role for corticosteroids in the early stages of aspiration pneumonitis, there is currently a lack of guidelines regarding the specific type, dosage, or route of corticosteroid administration.^[1,4,5,8]^ We report a postpartum woman who underwent general anesthesia during labor and subsequently developed acute aspiration pneumonitis after inhaling gastric contents, rapidly progressing to acute respiratory distress syndrome (ARDS). The patient was successfully treated with mechanical ventilation, fluid resuscitation, sedation, prophylactic antibiotics, and empirically administered a short course of high-dose corticosteroids. Through this case, we aim to discuss the potential role of corticosteroids in the management of aspiration pneumonitis, particularly during the early, noninfectious inflammatory phase.

## 2. Case report

A 27-year-old woman was transferred to our center due to rapidly progressing respiratory failure that began 2 hours after a cesarean section. She delivered at 37 + ^5^/_7_ weeks of gestation for cephalopelvic disproportion in a neighboring maternity hospital. Her medical history was unremarkable; she had no chronic illnesses, allergies, or previous respiratory complaints. Four hours before the operation, she drank 300 mL of milk.

The procedure started under epidural anesthesia, but was converted to general anesthesia with endotracheal intubation. Intraoperative blood loss was approximately 300 mL. During anesthesia, she aspirated gastric contents. She was extubated 2 hours postoperatively, yet within 1 hour developed acute dyspnea and hypoxemia (SpO_2_ = 82%–85% on 10 L/min oxygen via non-rebreathing mask) accompanied by a dry cough and no fever. She was promptly transferred to our hospital for further management. On arrival at the Emergency Department, the patient was conscious but agitated. Vital signs were: blood pressure = 140/80 mm Hg, heart rate = 130 beats/min, respiratory rate = 30 breaths/min with accessory-muscle use, temperature = 37°C, and SpO_2_ = 80% on 10 L/min oxygen. Chest auscultation revealed diffuse bilateral crackles without wheeze. The cesarean wound was clean and dry; the remainder of the examination was unremarkable.

**Figure 1.**
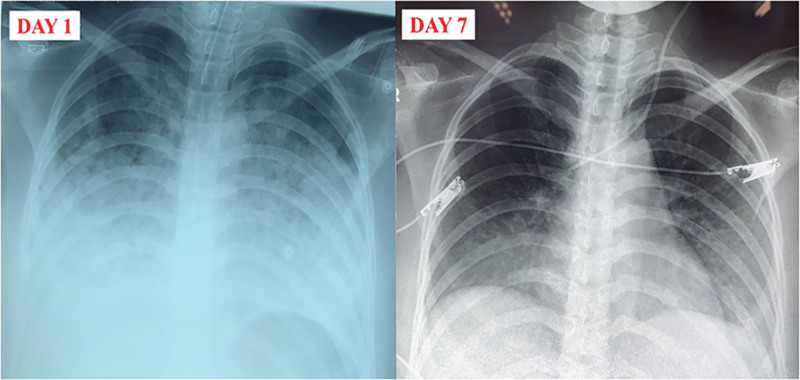
Sequential CXR illustrating the radiologic course in the postpartum patient. Day 1: diffuse bilateral air-space opacities consistent with chemical pneumonitis/early ARDS. Day 7: near-complete resolution and no tube following extubation on day 7. ARDS = acute respiratory distress syndrome.

**Figure 2.**
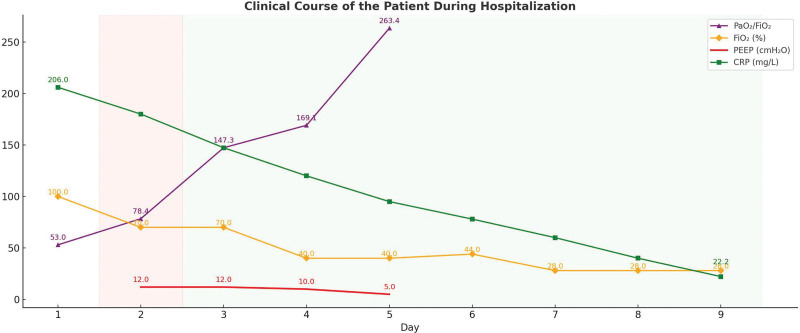
Clinical course of the patient during hospitalization. CRP = C-reactive protein, PEEP = positive end-expiratory pressure.

Four hours after the operation, her respiratory status deteriorated further. She was intubated and placed on invasive mechanical ventilation. Rapid sequence intubation was performed with IV propofol and sufentanil. After intubation, the patient had high spontaneous respiratory drive and ventilator asynchrony, so continuous deep sedation with midazolam and fentanyl by syringe pump was started. She then became comfortable and fully synchronous with the ventilator, and a continuous neuromuscular blocker was not required. The admission chest radiograph showed diffuse bilateral alveolar infiltrates more pronounced in the lower zones (Fig. 1). Arterial blood gas analysis on a FiO_2_ of 0.7 and a positive end-expiratory pressure (PEEP) of 12 cmH_2_O revealed severe hypoxemia with a PaO_2_/FiO_2_ ratio of 78.4 mm Hg, consistent with ARDS. ECG revealed sinus tachycardia (130 beats/min) without ST elevation or S_1_Q_3_T_3_ pattern, and transthoracic echocardiography was unremarkable, with no sign of right ventricular dysfunction. Initial laboratory data are summarized in Table 1. She was then transferred to the intensive care unit. The patient’s clinical course is presented in Figure 2.

**Table 1:** Laboratory results upon admission.

Tests	Results	Normal value
Hematology		
White-blood cell	**2.86**	4–11 G/L
% Neutrophil	**79.4**	45–75%
% Lymphocyte	**15.1**	20–40%
Hemoglobin	153	120–170 g/L
Platelet	**195**	200–400 G/L
Coagulation		
Prothrombin time	10.2	10–13 s
Activated partial thromboplastin time	**25.4**	26–37 s
Biochemistry		
C-reactive protein	**200.6**	<6 mg/L
Procalcitonin	0.25	<0.5 ng/mL
Aspartate transaminase	16	9–48 U/L
Alanine transaminase	33	5–49 U/L
Creatinine	0.61	0.7–1.5 mg/dL
D–dimer	**2585**	0–500 ng/mL
High-sensitivity troponin I	13.83	<15.6 pg/mL
NT-proBNP	**704.1**	<125 pg/mL
Arterial blood gas (after intubation, FiO₂ = 0.70)		
PO_2_	**54.9**	83–108 mm Hg
pH	**7.283**	7.35–7.45
pCO_2_	**49.6**	36–44 mm Hg
HCO_3_	23.7	22–26 mmol/L

Abnormal values are highlighted in bold.

Because aspiration pneumonitis was strongly suspected, we proceeded with early endotracheal intubation to secure the airway. An emergent bronchoscopy showed edematous, hyperemic airways without visible food particles or foreign bodies. Empirical intravenous meropenem and vancomycin were commenced, alongside lung-protective ventilation (tidal volume 6 mL/kg predicted body weight, plateau pressure ≤ 30 cmH_2_O), deep sedation, and prophylactic anticoagulation. Broad-spectrum empiric therapy was selected because we could not fully exclude early bacterial infection complicating the aspiration event, the patient was in life-threatening respiratory failure with a PaO_2_/FiO_2_ ratio of 78.4 mm Hg, and local microbiological data show frequent multidrug-resistant organisms, including extended-spectrum β-lactamase-producing *Enterobacterales* and methicillin-resistant *Staphylococcus aureus*, in critically ill patients. This initial approach provided coverage while awaiting culture results. On day 2, due to the risk of severe hypoxemia, methylprednisolone was administered at 1 mg/kg/day for 2 days. Oxygenation improved rapidly, allowing gradual reductions in FiO_2_ and PEEP. Blood, sputum, and bronchoalveolar lavage cultures were negative, and antibiotics were discontinued after 7 days. The patient was extubated on day 5 and discharged in good condition on day 10. A follow-up chest radiograph (Fig. 1) demonstrated resolution of infiltrates on day 7. She remained asymptomatic at a 2-week outpatient review.

## 3. Discussion

This case illustrates how aspiration of gastric contents during general anesthesia can precipitate a fulminant chemical pneumonitis that progresses to ARDS within hours. Our patient developed unusually rapid, severe ARDS (PaO_2_/FiO_2_ = 78.4) despite being previously healthy in the postpartum period. Management included lung-protective mechanical ventilation and early bronchoscopy. Broad-spectrum antibiotics were initiated because superimposed infection could not be excluded at presentation, although no microbiologically confirmed infection was identified. A key feature was our planned “start-reassess-stop” corticosteroid approach, methylprednisolone 1 mg/kg/day for 48 hours, followed by reassessment and early discontinuation when concern for superimposed infection emerged. Accordingly, the case provides a basis to discuss the controversial role of corticosteroids in aspiration pneumonitis, particularly focusing on their potential utility in modulating the intense, early, and presumably noninfectious, inflammatory phase that can lead to severe lung injury like ARDS. Our decision was guided by the biphasic pathophysiology of acid aspiration and contemporary evidence and guidance supporting systemic corticosteroids in ARDS, while actively balancing infection risk through close reassessment and early cessation when appropriate.

Mendelson first described acid aspiration pneumonitis during anesthesia in obstetric patients in 1946.^[9]^ Volumes as small as 0.3 mL/kg with a pH < 2.5 can damage the alveolar epithelium; food particles exacerbate injury even at higher pH.^[10–12]^ Importantly, experimental and clinical data support a biphasic mechanism of acid aspiration lung injury: an immediate epithelial/airway caustic insult followed by a neutrophil-dominant inflammatory phase beginning approximately 4 to 6 hours after the aspiration event.^[10,13]^ Pregnancy amplifies risk because most women have a gastric pH < 2.5, and about 60% harbor gastric volumes > 25 mL.^[14]^ Placental gastrin increases acid secretion, while the enlarging uterus and progesterone-mediated smooth muscle relaxation lower esophageal sphincter tone and delay gastric emptying. Intrapartum medications such as opioids and anticholinergics compound these effects. Additionally, airway anatomical changes make intubation more difficult, increasing the risk of aspiration during general anesthesia.^[15]^ Despite these factors, strict preventive protocols have reduced the incidence of aspiration pneumonitis.^[16,17]^ In a retrospective series of 48,609 cesarean deliveries, with 22,690 (46.7%) performed under general anesthesia, the incidence of aspiration pneumonitis was only 1 in 11,345.^[18]^ Nevertheless, when Mendelson syndrome does occur, it can precipitate severe lung injury and carries a poor prognosis if not recognized and managed promptly. The diagnosis of Mendelson syndrome requires a combination of high-risk profiles for aspiration, documented aspiration event when possible, rapidly progressing respiratory symptoms, and supportive clinical findings. A major challenge is that aspiration often goes unobserved, obliging clinicians to rely on risk factors while systematically ruling out alternative causes, such as cardiogenic pulmonary edema, pulmonary embolism, or amniotic fluid embolism.

In our patient, the anesthesiologist observed gastric aspiration during labor. This was followed by abrupt respiratory distress with hypoxemia, increased work of breathing, and new infiltrates on chest X-ray. Elevated C-reactive protein, low procalcitonin, and negative microbial cultures from bronchoalveolar lavage fluid and blood all favored a noninfectious etiology. Bronchoscopy further revealed edematous and hyperemic bronchial mucosa, consistent with acid-induced injury, while a normal transthoracic echocardiogram made cardiogenic pulmonary edema or pulmonary embolism unlikely, thereby confirming the diagnosis of aspiration pneumonitis. The lung injury rapidly progressed to ARDS, evidenced by worsening hypoxemia, bilateral infiltrates on chest X-ray, and a PaO_2_/FiO_2_ ratio < 300 mm Hg, presumably driven by an intense inflammatory response to the aspirated gastric acid.

A major challenge in managing aspiration syndromes is differentiating aspiration pneumonitis from aspiration pneumonia. Both result from inhalation of foreign material, yet their etiologies differ: pneumonitis is a sterile chemical injury caused by gastric acid, whereas pneumonia involves oropharyngeal secretions containing pathogens.^[4,19]^ Aspiration pneumonia typically presents with fever, productive cough, and gradual symptoms, while pneumonitis leads to rapid-onset respiratory distress. Although bacterial superinfection can occur, aspiration pneumonitis is initially noninfectious, and routine antibiotic use is not always necessary.^[1,4]^ However, there is no single gold standard to distinguish the 2 at presentation, and commonly used biomarkers such as procalcitonin are unreliable, in 1 study, procalcitonin levels did not differ significantly between aspiration pneumonitis and aspiration pneumonia.^[20]^ Consequently, especially in severe respiratory failure settings, clinicians frequently initiate empiric broad-spectrum antibiotics while awaiting culture data, despite no definitive evidence of infection, as occurred in our patient.^[21]^

Aspiration pneumonitis is best prevented rather than treated. General anesthesia should be avoided when possible; clear fluids are allowed until 2 hours preinduction, whereas milk or solids require 6 to 8 hours of fasting.^[6]^ Acid-neutralizing or pro-motility agents (such as antacids, H_2_-blockers, prokinetics) further lower risk by raising gastric pH and accelerating emptying.^[22,23]^ In our case, the patient consumed 300 mL of milk 4 hours before delivery, with no antacid prophylaxis. General anesthesia was chosen because of cephalopelvic disproportion. These circumstances likely increased her aspiration risk. Once aspiration is suspected, airway protection is critical: prompt endotracheal intubation, aggressive suctioning, and, when available, early bronchoscopy (<24 hours) to clear particulate matter. Recent data in ventilated patients show that such early bronchoscopy improves gas exchange, reduces secondary pneumonia, enables earlier antibiotic de-escalation, and may lower mortality.^[24–26]^

In our patient, we prioritized early endotracheal intubation rather than noninvasive ventilation (NIV). On presentation, she already met the Berlin criteria for severe ARDS, with PaO_2_/FiO_2_ = 78.4 mm Hg. Evidence supporting NIV in this setting is limited: a recent systematic review reported NIV failure in 48% of ARDS overall and 71% of severe ARDS, with many patients ultimately requiring intubation.^[27]^ NIV as first-line support in patients with PaO_2_/FiO_2_ < 150 mm Hg has also been associated with higher intensive care unit mortality compared with early invasive mechanical ventilation (LUNG SAFE cohort).^[28]^ A PaO_2_/FiO_2_ of 78.4 mm Hg, therefore, placed our patient in a very high-risk group for NIV failure. In addition, NIV can permit large, unmonitored patient-generated tidal volumes above lung-protective targets, raising concern for patient self-inflicted lung injury.^[29]^ Finally, intubation secured the airway in the setting of witnessed aspiration, as milklike material was visualized in the trachea, indicating residual gastric contents and ongoing aspiration risk, and allowed bronchoscopy, suctioning, and strict lung-protective ventilation with appropriate PEEP.

It is important to note that no guideline currently addresses aspiration pneumonitis as a sterile chemical injury distinct from aspiration pneumonia. Existing guidelines primarily discuss aspiration pneumonia as a bacterial process, whereas our patient’s presentation was dominated by chemical pneumonitis rapidly evolving into severe ARDS. Corticosteroids are considered for aspiration-induced lung injury because of their anti-inflammatory effects. Evidence, however, is controversial: several studies found little or no benefit, whereas others report clinical improvement.^[30,31]^ Gurganus reported 2 cases successfully managed with the HAT protocol (ascorbic acid, hydrocortisone 50 mg IV every 6 hours, and thiamin).^[32]^ A separate case of diesel fuel pneumonitis responded to methylprednisolone 60 mg IV daily plus empiric antibiotics.^[33]^ In a retrospective study by Zhao, methylprednisolone administered at an average dose of 1.14 mg/kg/day over 7.3 days was associated with improved respiratory parameters and reduced mortality, without a significant increase in adverse effects.^[5]^ Notably, there is no consensus regarding the optimal agent, dose, duration, or route.^[5,32,33]^ Because robust, disease-specific trials in aspiration pneumonitis are lacking, clinicians often extrapolate from evidence in ARDS more broadly. In moderate to severe ARDS, multiple randomized and observational studies have used systemic corticosteroids, given in various regimens, with lower 28-day mortality, shorter duration of mechanical ventilation, and reduced intensive care unit length of stay.^[34–36]^ Notably, the DEXA-ARDS trial (dexamethasone in established moderate to severe ARDS) demonstrated significant reductions in ventilator days and mortality.^[34]^ Meduri also reported benefit with methylprednisolone in early severe ARDS in a randomized trial.^[35]^ The 2024 Society of Critical Care Medicine and the 2024 American Thoracic Society guidelines both provide a conditional recommendation to administer systemic corticosteroids in adult ARDS. These guidelines also emphasize that optimal agent, dose, and duration remain uncertain, and they highlight clinically important adverse effects (hyperglycemia, glucocorticoid-induced muscle atrophy, or superinfection), supporting an individualized approach with close monitoring and reassessment.^[37,38]^

In this case, we decided to use corticosteroids for 2 main reasons. First, we recognized that ARDS is fundamentally an inflammatory process, and thus corticosteroids could help address the inflammation. Second, although there are no specific randomized controlled trials for chemical pneumonitis, international guidelines, such as those from the 2024 Society of Critical Care Medicine and 2024 American Thoracic Society clinical practice guidelines, suggest the use of corticosteroids in moderate to severe ARDS, which informed our approach. We started corticosteroids strategy with the biphasic inflammatory response to acid aspiration and current evidence for aspiration-related ARDS, while deliberately limiting duration to reduce the risk of adverse effects. Given the neutrophil-dominant phase beginning 4 to 6 hours after aspiration and the patient’s rapid progression to severe ARDS, we initiated a short, planned trial of intravenous methylprednisolone (1 mg/kg/day) with predefined reassessment and stopping criteria. Following initiation, FiO_2_ requirements fell, and oxygenation improved, but rising white-blood cell count and C-reactive protein raised concern for evolving superimposed infection, prompting steroid discontinuation after 2 days. Clinically, this case suggests that early airway protection with strict lung-protective ventilation and appropriate PEEP should be prioritized, and that early bronchoscopy, when feasible, may support diagnosis. Furthermore, the case also reinforces a stewardship-based approach to antimicrobials, with empiric antibiotics used when infection cannot be confidently excluded and de-escalated when cultures remain negative and clinical features favor chemical injury. Finally, in selected patients with early chemical aspiration-related moderate to severe ARDS, a carefully monitored, time-limited corticosteroid trial with predefined stopping criteria may be considered, with close surveillance for adverse effects and emerging infection.

## 4. Conclusion

Mendelson syndrome can progress rapidly, making timely diagnosis and treatment challenging. Corticosteroids may help reduce inflammation and improve respiratory function in early-phase chemical aspiration pneumonitis, especially when bacterial infection is not confirmed. In our case, early corticosteroid use was associated with rapid improvement, suggesting a potential benefit in selected patients. However, routine use remains controversial due to limited guidelines and possible side effects. More research is needed to clarify optimal indications, dosing, and duration.

## Author contributions

**Data curation:** An Le-Hoang, Khoa Nguyen-Dang, Huy Tran-Dinh, Ngoc Duong-Minh.

**Investigation:** An Le-Hoang, Khoa Nguyen-Dang, Huy Tran-Dinh.

**Methodology:** An Le-Hoang, Khoa Nguyen-Dang, Huy Tran-Dinh, Quoc-Khanh Tran-Le.

**Visualization:** An Le-Hoang, Khoa Nguyen-Dang.

**Conceptualization:** Khoa Nguyen-Dang, Ngoc Duong-Minh, Hanh-Duyen Bui-Thi.

**Formal analysis:** Khoa Nguyen-Dang, Quoc-Khanh Tran-Le.

**Project administration:** Khoa Nguyen-Dang, Ngoc Duong-Minh.

**Supervision:** Khoa Nguyen-Dang, Quoc-Khanh Tran-Le.

**Validation:** Khoa Nguyen-Dang, Quoc-Khanh Tran-Le.

**Funding acquisition:** Ngoc Duong-Minh.

**Software:** Ngoc Duong-Minh.

**Resources:** Hanh-Duyen Bui-Thi.

**Writing – original draft:** An Le-Hoang, Khoa Nguyen-Dang, Huy Tran-Dinh, Quoc-Khanh Tran-Le, Ngoc Duong-Minh, Hanh-Duyen Bui-Thi.

**Writing – review & editing:** An Le-Hoang, Khoa Nguyen-Dang, Huy Tran-Dinh, Quoc-Khanh Tran-Le, Ngoc Duong-Minh, Hanh-Duyen Bui-Thi.
